# Severe brain injury ICU outcomes are associated with Cranial-Arterial Pressure Index and noninvasive Bispectral Index and transcranial oxygen saturation: a prospective, preliminary study

**DOI:** 10.1186/cc5097

**Published:** 2006-11-14

**Authors:** C Michael Dunham, Kenneth J Ransom, Clyde E McAuley, Brian S Gruber, Dev Mangalat, Laurie L Flowers

**Affiliations:** 1 Trauma/Critical Care Services, St Elizabeth Health Center, Youngstown, OH 44501, USA

## Abstract

**Introduction:**

The purpose of this study was to determine if noninvasive transcranial oxygen saturation (StcO_2_) and Bispectral Index (BIS) correlate with severe traumatic brain injury intensive care unit (ICU) outcomes.

**Methods:**

This is a prospective observational study. Values of intracranial pressure (ICP), mean arterial pressure (MAP), BIS, and StcO_2 _were recorded hourly for the first six, post-injury days in 18 patients with severe brain injury. Included in the analyses was the Cranial-Arterial Pressure (CAP) Index, which is ICP/(MAP - ICP).

**Results:**

After 1,883 hours of data were analyzed, we found that StcO_2 _and BIS are associated with survival, good neurological outcome, ICP ≤20, cerebral perfusion pressure (CPP) ≥60, and CAP index ≤0.30 (*p *≤ 0.001). Survival and good outcome are independently associated with BIS ≥60, StcO_2 _≥70, and ICP ≤20 (*p *< 0.0001). BIS ≥60 or StcO_2 _≥70 is associated with survival, good outcome, CPP ≥60, ICP ≤20, CAP index ≤0.30, and fewer ICP interventions (*p *< 0.0001). With BIS ≥60 or StcO_2 _≥70, the rate of CPP ≥60 is 97.2% and the rate of ICP≤ 25 is 97.1%. An increased CAP index is associated with death, poor neurological outcome, and increased ICP interventions (*p *< 0.0001). With CAP index >0.25, MAP is not related to ICP (*p *= 0.16).

**Conclusion:**

Numerous significant associations with ICU outcomes indicate that BIS and StcO_2 _are clinically relevant. The independent associations of BIS, StcO_2_, and ICP with outcomes suggest that noninvasive multi-modal monitoring may be beneficial. Future studies of patients with BIS ≥60 or StcO_2 _≥70 will determine if select patients can be managed without ICP monitoring and whether marginal ICP can be observed. An increased CAP index is associated with poor outcome.

## Introduction

The primary clinical objective after severe brain trauma is to prevent secondary injury, a common sequel to the primary, mechanical impact. The concept is to prevent cerebral hypoxia by maintaining sufficient oxygen delivery to meet the oxidative metabolic needs of the intracranial neural tissues. This implies that cerebral blood flow, arterial oxygen saturation, and hemoglobin concentration in a specific patient need to be adequate.

Intracranial pressure (ICP) and cerebral perfusion pressure (CPP) (mean arterial pressure (MAP) - ICP) monitoring is recommended for severe brain injury. There are several limitations of ICP and CPP monitoring: the ICP device is invasive and insertion requires rigorous training [1,2]; distinct ICP and CPP target recommendations are uncertain [3,4]; CPP is not equivalent to cerebral blood flow [5]; and, additionally, arterial oxygen content (arterial oxygen saturation (SaO_2_) and hemoglobin) and oxidative cerebral tissue needs, relative to oxygen delivery, are not intrinsic components of ICP and CPP.

Near-infrared oximetry provides a noninvasive method for measuring transcranial oxygen saturation (StcO_2_). StcO_2 _estimates regional cerebral capillary/venous oxygen saturation [6-8] StcO_2 _monitoring provides an opportunity to determine whether cerebral cortical oxygen delivery is adequate to meet cellular oxidative needs. Dunham and colleagues have shown that cerebral oximetry values correlate with outcomes and CPP following severe brain injury [9]. These findings have been corroborated by others [10].

A repertoire of electrical activity continuously emanates from the superficial cerebral cortex and can be displayed on an electroencephalogram (EEG). EEG tracings have been shown to be variably altered by sedatives, hypoxia, hypercarbia, ischemia, and intracranial hypertension [11]. The noninvasive, Bispectral Index (BIS) monitor creates a computer-processed summary of EEG brain wave activity [12]. The algorithm generates an ordinal number that rates level of hypnosis during anesthesia. Although BIS values have been shown to correlate with some intensive care unit (ICU) conditions, documented experience with severe brain injury is limited.

CPP monitoring is an attempt to estimate global cerebral blood flow. StcO_2 _monitoring assesses frontal cerebral cortical oxygen extraction (the relationship between oxygen delivery and consumption). BIS values are influenced by frontal cortical electrical activity. The study purpose is to determine the relationships between StcO_2 _and BIS values in severe brain injury and ICU outcomes (survival, discharge Glasgow Coma Scale (GCS) score, ICP, CPP, and interventions to lower ICP).

## Materials and methods

### Patient characteristics

Patients were considered for study entry if they had blunt traumatic head injury, initial GCS score ≤ 8, brain computed tomography (CT) scan that demonstrated a hemorrhagic lesion, age between 18 and 65 years, and an ICP monitor inserted within 24 hours post-injury. A CT hemorrhagic lesion (intracranial hemorrhage) was defined as the presence of an epidural hematoma, subdural hematoma, cerebral contusion, cerebral hemorrhage, or subarachnoid hemorrhage. Patients were excluded if there was pre-hospital cardiac arrest, near-brain-death clinical findings after resuscitation, pre-existing medical coagulopathy, or a body mass index ≥ 35 kg/m^2^. The Institutional Review Board for human investigations approved the study.

### Patient monitoring

BIS and StcO_2 _monitoring began when the ICP device was inserted and study consent was obtained. Each hour, ICP, MAP, StcO_2_, and BIS were monitored and recorded by the nursing staff. Cerebrospinal fluid (CSF) aspiration and mannitol administration were recorded hourly.

StcO_2 _was measured with the INVOS 4100 system (Somanetics Corporation, Troy, MI, USA). Self-adhesive skin patches, which contain a near-infrared light-emitting diode and two photodiode detectors to measure returning scattered light intensities, were applied to the patient's left and right forehead. The skin patches were connected to cables that communicate with a computer and a near-infrared light generator. Harmless near-infrared light is generated by the light-emitting diode. Photons easily pass through scalp and bone tissue and enter the cerebral cortex. Photons are scattered back to the two detectors. The detector near the emitting-diode measures photons in the superficial tissues (scalp and bone), whereas the far detector includes photons from the deep tissues (scalp, bone, and cerebral cortex). Hemoglobin molecules within capillary red blood cells are measured by each detector at the wavelengths of 730 (deoxyhemoglobin) and 810 (total hemoglobin) nanometers. The signal difference between the near and far detectors allows a calculation of regional capillary/venous oxygen saturation in the cerebral cortex. The oxygen saturation values reflect the balance between cerebral cortical oxygen delivery and consumption. This information is converted to a digital format and oxy-hemoglobin saturation is derived from these values. The StcO_2 _values are then displayed in real time on the computer screen. The mean value for the left and right sides was computed.

The noninvasive, A-2000 Bispectral Index XP Monitoring System (Aspect Medical Systems, Inc., Newton, MA, USA) continuously processes raw EEG signals to produce a single number, or BIS. BIS was designed to correlate with hypnotic clinical endpoints (sedation, lack of awareness, and memory) in order to track changes in the effects of anesthetics on the brain. The BIS correlates with the patient's level of hypnosis, where 100 indicates that the patient is awake and 0 represents a flat line EEG. The forehead sensor transmits EEG signals to the digital signal converter. The converter amplifies and digitizes these signals, then sends them to the monitor. The monitor software filters the data, analyzes it for artifacts, and processes it using digital signal processing techniques. The output from a multivariate discriminate analysis quantifies the overall bispectral properties (frequency, power, and phase) throughout the entire frequency range. The self-adhesive skin patch was randomly applied to the patient's left or right forehead. One side was selected whenever the opposite side had soft tissue injury.

### Patient interventions

Full-time surgical intensivists (four) and neurosurgeons (three) managed all patients and ordered interventions based on hourly ICP and CPP values. The hourly StcO_2 _and BIS values did not influence treatment decisions.

Routine clinical targets included: isotonic fluid administration at maintenance rates, hemoglobin >10 g/dL, SaO_2 _>92%, arterial carbon dioxide partial pressure (PaCO_2_) 35 to 42 torr, MAP 80 to 90 torr, head of bed elevation (15 to 30 degrees), euthermia, CPP ≥60 torr, euvolemia or mild hypervolemia, cardiac index ≥3.0 L/min/m^2^, serum osmolality ≥290 mOsm/kg, and serum lactate ≤2.5 mmol/L. Primary interventions for patients with ICP >20 torr included: brain CT scan to detect surgical lesions and the need for craniotomy, sedation when MAP ≥85 torr, CSF drainage, neuromuscular blockade for motor hyperactivity uncontrolled by sedatives or sedative-induced hypotension, mannitol (if serum osmolality <320 mOsm/kg or earlier, if cerebral edema was present), diuretics (for hypo-osmolar serum and/or hypervolemia), and modest hyperventilation (PaCO_2 _31 to 34 torr).

Secondary interventions for recalcitrant intracranial hypertension included: brain CT scan to detect surgical lesions that require a craniotomy, alpha agonist (dopamine (>8 μg/kg/minute), phenylephrine, or norepinephrine) to elevate MAP to a supranormal level, hypothermia, aggressive hyperventilation, barbiturate coma, and decompressive craniectomy. Interventions for systemic arterial hypotension included: for obvious vasodilation (capillary nail bed hyperemia or decreased systemic vascular resistance index), afterload augmentation with an alpha agonist and discontinuance of sedatives; for obvious hypovolemia (low central venous pressure or pulmonary artery occlusion pressure, low cardiac index, or fluid input much less than fluid output), fluid-bolus administration (250 mL of normal saline over 20 minutes), pitressin for diabetes insipidus, or red blood cells for hemoglobin <10 gm/dL; and, for impaired cardiac contractility (cardiac index <3.5 L/min/m^2^, or increased lactate and pulmonary artery occlusion pressure >15 torr), inotropic support. When the etiology was unclear, combinations of the above recommendations were used.

### Data collection

General information included patient age, gender, Injury Severity score, first-24-hour intracranial CT scan results (epidural hematoma, subdural hematoma, cerebral contusion or hematoma, midline shift >3 mm, abnormal mesencephalic cisterns, subarachnoid hemorrhage), brain Abbreviated Injury Scale score, initial GCS score, need for craniotomy, mortality outcome, and hospital discharge GCS score. Patients were determined to have a good neurological outcome if the hospital discharge GCS score was 9 to 15. Poor neurological outcome was assigned when a patient died or had a hospital discharge GCS score of 3 to 8.

The ICP, MAP, BIS, and StcO_2 _values were recorded hourly for each of the first six post-injury days. If the ICP device was removed prior to the sixth day, data collection was terminated. Day and hour values represented the period of time that had elapsed since the date and time of each patient's injury. Yes or no values were recorded for CSF drainage (≥5 mL in past one hour) and mannitol administration (given within the previous two hours). An intervention to lower ICP was considered as yes for a given hour if CSF was drained or mannitol was administered.

### Cranial-Arterial Pressure index

During preliminary data analyses the ICP to CPP ratio (ICP/(MAP - ICP)) was found to be highly discriminate for surviving and non-surviving patients. This relationship, created by the authors, is referred to as the Cranial-Arterial Pressure (CAP) Index and is included in multiple analyses.

### Statistical analysis

Data entry and preliminary data analyses were conducted using Epi Info version 6.04d (Centers for Disease Control and Prevention, Atlanta, GA, USA). Data were exported from Epi Info into SAS for windows version 8.00 (SAS statistical software, Cary, NC, USA) for statistical analysis. The Shapiro-Wilk Test is used to determine whether the data are normally distributed. Measurements are reported as the mean value ± the standard deviation. Group frequencies are compared with the Chi-square test. Comparison of inter-group continuous variables is by *t*-test. Relationship assessment between two continuous variables is by Pearson correlation coefficient. Multivariate logistic regression analysis is used to evaluate the effect of independent continuous or dichotomous variables (for example, CPP ≥60, ICP ≤20, BIS, and StcO_2_) on dichotomous dependent variables (for example, mortality, neurological outcome). Level of statistical significance was set at *p *< 0.05 for all tests.

## Results

The study includes 18 consecutive patients and was conducted from July 2005 until May 2006. There are 1,883 concurrent, hourly observations of ICP, CPP, BIS and StcO_2 _values. Injury characteristics are displayed in Table 1. The data are normally distributed (MAP - W = 0.99; ICP - W = 0.89; CPP - W = 0.98; BIS - W = 0.99; StcO_2 _- W = 0.99; *p *< 0.0001 for all variables). Surviving and good neurological outcome patients have increased CPP, StcO_2_, and BIS and decreased ICP and CAP Index (Table 2). ICP, CPP, BIS and StcO_2 _rates are: ICP ≤20 = 84.9% (1,598); CPP ≥60 = 93.9% (1,768); BIS ≥60 = 30.9% (582); and StcO_2 _≥70 = 50.4% (949). Survival is independently associated with ICP ≤20, BIS ≥60, and StcO_2 _≥70 (*p *< 0.0001). Good neurological outcome is independently associated with ICP ≤20, BIS ≥60, and StcO_2 _≥70 (*p *< 0.0001). Survival is independently associated with CPP ≥60, BIS ≥60, and StcO_2 _≥70 (*p *< 0.0001). Good neurological outcome is independently associated with CPP ≥60, BIS ≥60, and StcO_2 _≥ 70 (*p *< 0.0001). Interactive variables are either not statistically significant or have no impact on model predictability.

StcO_2 _and BIS have an inverse association with ICP and CAP Index, and a direct association with CPP (Table 3). StcO_2 _has a direct association with BIS (Table 4). Combined BIS and StcO_2 _rates are: BIS ≥60 or StcO_2 _≥70 = 61.2% (1,152); and BIS <60 and StcO_2 _<70 = 38.8% (731). BIS ≥60 or StcO_2 _≥70 is associated with survival, good neurological outcome, CPP ≥60, ICP ≤20, CAP Index ≤0.30, and less interventions to lower ICP (Table 5). The majority of observations for surviving and good neurological outcome patients have BIS ≥60 or StcO_2 _≥70. The majority of observations for dying and poor neurological outcome patients have BIS <60 and StcO_2 _<70. With BIS ≥60 or StcO_2 _≥70, the rate for CPP ≥60 is 97.2% (95% confidence interval (CI) 96.1 to 98.0), the rate for ICP ≤20 is 90.8% (95% CI 89.0 to 92.3%), and the rate for ICP ≤25 is 97.1% (95% CI 96.0 to 98.0%).

An increasing CAP Index indicates a modest reduction in MAP and substantial increase in ICP (Table 6). As the CAP Index increases, the magnitude of change in this variable is much greater in comparison to the changes in MAP, ICP, and CPP. The CAP Index is increased with death, poor neurological outcome, and need for interventions to lower ICP (Table 7). The CAP Index has the following correlation coefficients: ICP - r = 0.70, *p *< 0.0001; MAP - r = -0.18, *p *< 0.0001; and CPP - r = -0.55, *p *< 0.0001. Survival is independently associated with CAP Index and CPP (*p *= 0.0001). Good neurological outcome is independently associated with CAP Index (*p *= 0.0001), but not CPP (*p *= 0.29). The need for interventions to lower ICP (mannitol and/or CSF aspiration) is independently associated with CAP Index and ICP (*p *= 0.0001). The need for interventions to lower ICP (mannitol and/or CSF aspiration) is independently associated with CAP Index (*p *= 0.0001), but not CPP (*p *= 0.08). When the CAP Index is >0.25 (*n *= 365; MAP - 91.0 ± 12.0; ICP - 27.1 ± 9.1; CPP - 64.0 ± 14.6), there is no relationship between MAP and ICP (r = 0.07; *p *= 0.16)

## Discussion

This is a prospective study evaluating 18 consecutive patients with severe brain injury. It includes 1,883 hourly concurrent observations of ICP, CPP, BIS and StcO_2_. The study findings indicate that BIS and StcO_2 _are clinically relevant variables, because they are associated with ICU outcomes (survival, hospital discharge GCS, ICP, CPP, and interventions to lower ICP). Surviving patients and patients with good neurological outcome have higher BIS and StcO_2 _values. BIS and StcO_2 _are inversely related to ICP and CAP Index and directly associated with CPP. BIS and StcO_2 _have a positive relationship. The data suggest that BIS, StcO_2_, ICP, and CPP are related, but distinct indices of outcome.

ICP and CPP monitoring have substantial limitations. There are insufficient data to support a treatment standard for ICP treatment threshold, a principle that reflects a high degree of clinical certainty [3]. ICP treatment should be "initiated at an upper threshold of 20 to 25 mmHg", a principle that reflects a moderate degree of clinical certainty [3]. The moderate degree of clinical certainty, the nebulous recommendation to initiate treatment, and the ICP range indicate that a precise ICP endpoint has not been realized. There are insufficient data to support a treatment standard for a targeted CPP, a principle that reflects a high degree of clinical certainty [4]. CPP should be maintained at a minimum of 60 mmHg, a principle that reflects a moderate degree of clinical certainty. Further, ICP devices are invasive and insertion requires expertise [1,2]. Further indication that ICP and CPP targets are unclear is the controversy between CPP versus ICP management [13-15] Additionally, CPP does not equate to cerebral blood flow [5]. Finally, arterial oxygen content (SaO_2 _and hemoglobin) and oxidative cerebral tissue needs, relative to oxygen delivery, are not components of ICP and CPP. These ICP and CPP constraints suggest that additional monitoring techniques are needed.

The multiple associations of BIS and StcO_2 _with survival, neurological outcome, ICP, and CPP suggest that BIS and StcO_2 _are clinically discriminate parameters. Surviving patients have decreased ICP, increased CPP, decreased CAP Index, increased StcO_2_, and increased BIS. Good neurological outcome patients also have decreased ICP, increased CPP, decreased CAP Index, increased StcO_2_, and increased BIS. Variation in StcO_2 _and BIS are associated with changes in ICP and CPP. Such statistical associations support the validity of StcO_2 _and BIS and their potential clinical utility.

The independent association of BIS, StcO_2_, and ICP with outcomes indicates that BIS, StcO_2_, and ICP data are complementary. BIS, StcO_2_, ICP, and CPP are related, but distinct indices of outcome with severe brain injury. Survival is independently associated with BIS, StcO_2_, ICP, and CPP. Good neurological outcome is also independently associated with BIS, StcO_2_, ICP, and CPP. Other studies indicate that supplemental monitoring in severe brain injury is associated with clinical benefits. Cruz [16] showed that managing cerebral extraction of oxygen in conjunction with CPP is associated with better neurological outcomes than when CPP treatment alone is used. Other patients with severe brain injury and receiving multi-modal monitoring have improved survival when compared to ICP and CPP monitoring [17]. The study and literature findings are in support of severe brain injury multi-modal monitoring.

The study findings indicate that, when ICP increases or CPP decreases, there is cerebral hypoxia and altered brain wave patterns. With increased ICP or decreased CPP, StcO_2 _is reduced. As well, increased ICP or decreased CPP are associated with a reduction in BIS. Increased BIS is associated with increased StcO_2_.

BIS ≥ 60 or StcO_2 _≥ 70 suggest that patients with severe brain injury are stable. A BIS ≥60 or StcO_2 _≥70 are associated with survival, good neurological outcome, increased CPP, decreased ICP, decreased CAP Index, and decreased interventions to lower ICP. With a BIS ≥60 or StcO_2 _≥70, ICP and CPP are likely to be acceptable. A BIS ≥60 or StcO_2 _≥70 indicate there is a high probability of an acceptable CPP (CPP ≥60, 97.2% rate) and ICP (ICP ≤20, 90.8% rate; ICP ≤25, 97.1% rate).

Our previous investigation included 3,722 hourly observations of StcO_2 _in patients with severe brain injury [9]. This study also demonstrates that StcO_2 _is associated with survival and CPP in a similar patient cohort. These findings have been validated by other investigators [10]. In severe brain injury, BIS values correlate with recovery of consciousness [18], the raw EEG during barbiturate coma [19], and brain death [20]. In patients with variable degrees of brain injury, BIS correlates with positive brain CT scan and neurological outcome [21].

Future studies may determine if select patients with a BIS ≥60 or StcO_2 _≥70 can be managed without ICP monitoring. Potential candidates are blunt trauma patients with intracranial hemorrhage, GCS score 6 to 8, no need for emergency craniotomy, differentiated gray-white matter, no significant midline shift, patent basal cisterns, patent sulci, and reactive, symmetric pupils. With a BIS ≥60 or StcO_2 _≥70, an ICP monitor may not be necessary. With a BIS <60 and StcO_2 _<70 an ICP monitor is indicated. Other patients who may benefit from BIS and StcO_2 _monitoring without ICP monitoring are blunt trauma patients with intracranial hemorrhage, GCS score 9 to 12, and the need for mechanical ventilation and sedation.

Additional investigations may prove that a marginal ICP in blunt trauma patients with intracranial hemorrhage and GCS score 3 to 8 does not need to be lowered with BIS ≥60 or StcO_2 _≥70. Specifically, when ICP is 15 to 20 mmHg and BIS ≥60 or StcO_2 _≥70, interventions to lower ICP may be unnecessary. However, with BIS <60 and StcO_2 _<70, interventions to lower ICP or increase CPP should be considered.

The CAP Index is a parameter that was identified during this study. However, no other study, to our knowledge, has described such a relationship. CAP Index is a relationship that readily classifies patients according to neurological outcomes. An elevated CAP Index indicates a modest reduction in MAP and substantial increase in ICP. When comparing the surviving and nonsurviving patients, the mean difference is much greater for the CAP Index than it is for ICP, CPP, StcO_2_, and BIS. Similar relative differences are noted when comparing patients with good neurological outcome and bad outcome. An increased CAP Index is associated with death, poor neurological outcome, and increased interventions to lower ICP. CAP Index is correlated with, but not identical to, ICP, MAP, and CPP. CAP Index has an additional association with survival, good neurological outcome, and lack of need for interventions to lower ICP, independent of ICP and CPP. These findings suggest that the CAP Index is a distinct, interactive parameter. When the CAP Index is increased, there is no relationship between MAP and ICP. When there is no relationship between MAP and ICP, increasing MAP typically will not increase ICP [15,22,23] Thus, when the CAP Index is increased and ICP cannot be reduced, increasing MAP may improve CPP. This implication needs to be tested.

There are several study limitations. A larger group of patients needs to be evaluated to confirm the observations in this study. Severe forehead, soft tissue injury may prohibit BIS or cerebral oximetry sensor application or alter the BIS or StcO_2 _values. Bilateral frontal lobe contusions may alter the StcO_2 _values, thus impeding their clinical interpretation. A frontal subdural hematoma may interfere with BIS or StcO_2 _values. Discharge GCS score status was included, as one of several measures, to assess ICU outcomes. Admission GCS score was 3 to 8 for all patients, indicating severe neurological impairment. A discharge GCS score of 3 to 8 would indicate relatively poor neurological outcome. A discharge GCS score of 9 to 15 would indicate an improvement and a relatively good neurological outcome, when compared to admission. Because of the above, discharge GCS score was dichotomized as a relative indication of ICU outcome. However, quality of life and Glasgow Outcome score assessment at six months may be a more relevant indication of neurological outcome. These outcomes need to be compared with post-injury BIS and StcO_2 _values. The study focuses on severe traumatic brain injury. CT scan evidence of intracranial hemorrhage was a study inclusion requirement, because it suggests a history of mechanical brain trauma. Severe cognitive impairment due to mechanical brain injury can occur without intracranial hemorrhage, although this is relatively uncommon. With post-traumatic severe cognitive impairment, the absence of intracranial hemorrhage suggests that hypoxemia or shock may be the primary pathology. The study does not address patients with hypoxic encephalopathy, medical subarachnoid hemorrhage, and diffuse axonal injury. A larger group of patients needs to be studied to determine the impact of individual brain pathology on outcomes and BIS and StcO_2 _values. Prospective trials need to be performed to assess the hour-to-hour treatment implications of BIS and StcO_2 _values. The CAP Index, to our knowledge, has never been described in the literature. Additional investigations need to be conducted to define its therapeutic implications. Further studies are required to determine if the prognostic implications found in this study can be corroborated.

## Conclusion

CPP, BIS, and StcO_2 _monitoring are intended to assess global intracranial blood flow, regional cerebral cortical function, and local cortical oxygen extraction, respectively. The associations of BIS and StcO_2 _with ICU outcomes (survival, neurological outcome, ICP, CPP, CAP Index, and interventions to lower ICP) indicate that BIS and StcO_2 _are clinically discriminate parameters. The independent associations of BIS, StcO_2_, and ICP or CPP with outcomes indicate that BIS, StcO_2_, and ICP values are complementary. Apropos, the noninvasiveness of BIS and StcO_2 _is appealing. ICP and CPP monitoring are limited by non-distinct targets and need for expertise with monitor insertion. Study findings indicate that cerebral hypoxia occurs and brain wave patterns are altered when ICP increases, CPP decreases, or CAP Index increases. BIS ≥60 or StcO_2 _≥70 suggest that patients with severe brain injury are likely to have an acceptable ICP and CPP. Future studies will define the role for BIS and StcO_2 _monitoring with traumatic brain injury. They will determine if select patients with BIS ≥60 or StcO_2 _≥70 can be managed without ICP monitoring. Such investigations may prove that an ICP of 16 to 25 mmHg does not need to be lowered with BIS ≥60 or StcO_2 _≥70. An increased CAP Index is a harbinger of poor outcome. Further studies may show that, when the CAP Index is increased and ICP cannot be reduced, raising MAP will enhance CPP.

## Key messages

• Noninvasive BIS and StcO_2 _values are clinically relevant with severe brain injury.

• ICP, BIS, and StcO_2 _values provide complementary information.

• When ICP increases or CPP decreases, there is cerebral hypoxia and altered brain wave patterns.

• Future studies will determine the therapeutic and diagnostic benefit of BIS and StcO_2 _monitoring.

• CAP Index is a discriminate relationship between ICP and MAP that has prognostic and therapeutic implications.

## Abbreviations

BIS = Bispectral Index; CAP = Cranial-Arterial Pressure; CI = confidence interval; CPP = cerebral perfusion pressure; CSF = cerebrospinal fluid; CT = computed tomography; EEG = electroencephalogram; GCS = Glasgow Coma Scale; ICP = intracranial pressure; ICU = intensive care unit; MAP = mean arterial pressure; StcO_2 _= transcranial oxygen saturation.

## Competing interests

The authors declare that they have no competing interests.

## Authors' contributions

CMD conceived and coordinated the study, and was involved in the study organization, data collection, analysis, and interpretation, and manuscript draft and revisions. KJR was involved in the study organization, data analysis and interpretation, and manuscript draft and revisions. CEM participated in the study organization, data interpretation, and manuscript draft and revisions. BSG contributed to the study organization, data interpretation, and manuscript draft and revisions. DM was involved in the data collection, analysis, and interpretation, and manuscript revisions. LF contributed to the study organization, data collection and interpretation, and manuscript revisions. All authors read and approved the final manuscript.

## Figures and Tables

**Table 1:** Injury characteristics of 18 consecutive patients with severe traumatic brain injury

	Mean/number	SD/percent
Age	34.3	13.1
Male	11	61.1 percent
Female	7	38.9 percent
Weight (kg)	80.3	15.7
Height (cm)	175.4	8.1
ISS	37.2	8.9
Admission GCS	4.4	1.9
Brain AIS score	4.9	0.2
Admission lactate	4.5	2.7
Admission base deficit	-6.8	5.1
Admission glucose	203.7	80.3
RBC units	1.4	3.3
Admission WBC count	19.6	7.5
Craniotomy	8	44.4 percent
Bone flap removal	6	33.3 percent
EDH	2	11.1 percent
SDH	7	38.9 percent
Cerebral hemorrhage	11	61.1 percent
Cerebral edema	3	16.7 percent
Midline shift	8	44.4 percent
Abnormal cisterns	10	55.6 percent
SAH	12	66.7 percent
Discharge GCS	9.7	3.7
Lived	16	88.9 percent
Died	2	11.1 percent
Good neurological outcome	14	77.8 percent
Poor neurological outcome	4	22.2 percent

AIS, Abbreviated Injury Scale; EDH, epidural hematoma; GCS, Glasgow Coma Scale; ISS, Injury Severity score; RBC, red blood cell; SAH, subarachnoid hemorrhage; SD, standard deviation; SDH, subdural hematoma; WBC, white blood cell.

**Table 2:** Surviving and good neurological outcome patients have increased CPP, StcO_2_, and BIS and decreased ICP and CAP Index

	Hours	ICP	CPP	CAP Index	StcO_2_	BIS
Live	1,683	11.8 ± 6.1	81.5 ± 13.5	0.15 ± 0.10	70.0 ± 9.3	51.1 ± 16.5
Die	200	30.0 ± 11.1	66.6 ± 21.6	0.63 ± 0.79	61.0 ± 5.2	47.8 ± 12.9
*p *value		<0.0001	<0.0001	<0.0001	<0.0001	0.002
						
Good outcome	1,479	11.8 ± 6.4	82.1 ± 13.8	0.15 ± .10	71.2 ± 9.1	52.9 ± 16.8
Poor outcome	404	20.8 ± 12.3	72.0 ± 17.7	0.39 ± .61	61.2 ± 5.4	44.4 ± 13.0
*p *value		<0.0001	<0.0001	<0.0001	<0.0001	<0.0001

BIS, Bispectral Index; CAP Index, Cranial-Arterial Pressure Index (ICP/(MAP - ICP)); CPP, cerebral perfusion pressure; ICP, intracranial pressure; MAP, mean arterial pressure; StcO_2_, transcranial oxygen saturation.

**Table 3:** StcO_2 _and BIS have an inverse association with ICP and CAP Index and a direct association with CPP

	StcO_2_					BIS				
	≥70 (percent)	<70 (percent)	OR	95 percent CI	*p *value	≥60 (percent)	<60 (percent)	OR	95 percent CI	*p *value

ICP >20	9.8	20.6	0.42	0.32–0.55	<0.0001	6.5	19.0	0.30	0.21–0.43	<0.0001
CAP Index >0.30	7.9	17.7	0.40	0.30–0.54	<0.0001	5.3	16.1	0.29	0.19–0.44	<0.0001
CPP ≥ 60	97.5	90.3	4.2	2.6–6.8	<0.0001	97.8	92.2	3.7	2.0–7.0	<0.0001

BIS, bispectral index; CAP Index, Cranial-Arterial Pressure Index (ICP/(MAP - ICP)); CI, confidence intervals; CPP, cerebral perfusion pressure; ICP, intracranial pressure; OR, odds ratio; StcO_2_, transcranial oxygen saturation.

**Table 4:** StcO_2 _and BIS have a direct association

	BIS ≥ 60 (percent)	BIS <60 (percent)	OR	95 percent CI	*p *value
StcO_2 _≥ 70	39.9	60.1	2.4	2.0–3.0	<0.0001
StcO_2 _<70	21.7	78.3			

BIS, bispectral index; CI, confidence intervals; OR, odds ratio; StcO_2_, transcranial oxygen saturation.

**Table 5:** BIS ≥ 60 or StcO_2 _≥ 70 are associated with survival, good neurological outcome, CPP, ICP, CAP Index, and ICP interventions

	BIS ≥ 60/StcO_2 _≥ 70 (percent)	BIS <60/StcO_2 _<70 (percent)	OR	95 percent CI	*p *value
Live	67.7	32.3	30.1	16.6–55.8	<0.0001
Die	6.5	93.5			
Good neurological outcome	74.8	25.2	23.1	16.4–32.5	<0.0001
Poor neurological outcome	11.4	88.6			
CPP ≥ 60	97.2	88.7	4.5	2.9–7.0	<0.0001
CPP <60	2.8	11.4			
ICP ≤ 20	90.8	75.5	3.2	2.4–4.2	<0.0001
ICP >20	9.2	24.5			
CAP Index ≤ 0.30	92.4	79.1	3.2	2.4–4.3	<0.0001
CAP Index >0.30	7.6	20.9			
No mannitol/CSF drainage	85.7	62.4	3.6	2.9–4.5	<0.0001
Mannitol/CSF drainage	14.3	37.6			

BIS, Bispectral Index; CAP Index, Cranial-Arterial Pressure Index (ICP/(MAP - ICP)); CI, confidence intervals; CPP, cerebral perfusion pressure; CSF, cerebrospinal fluid; ICP, intracranial pressure; OR, odds ratio; StcO_2_, transcranial oxygen saturation.

**Table 6:** An increasing CAP Index indicates a modest reduction in MAP and substantial increase in ICP

CAP Index range	Number	Percent	MAP	ICP	CPP	CAP Index
0.01–0.10	521	27.7	95.7	5.2	90.4	0.06
0.11–0.20	745	39.6	93.3	12.1	81.3	0.15
0.21–0.30	377	20.0	93.7	18.1	75.6	0.24
0.31–0.40	106	5.6	91.2	23.2	68.0	0.34
> 0.40	134	7.1	89.4	36.8	52.5	0.87

CAP Index, Cranial-Arterial Pressure Index (ICP/(MAP - ICP)); CPP, cerebral perfusion pressure; ICP, intracranial pressure; MAP, mean arterial pressure.

**Table 7:** The CAP Index is increased with death, poor neurological outcome, and need for interventions to lower ICP

	CAP Index	*p *value
Die	0.63	<0.0001
Live	0.15	
Poor neurological outcome	0.39	<0.0001
Good neurological outcome	0.15	
Mannitol/CSF drainage	0.27	<0.0001
No mannitol/CSF drainage	0.18	

CAP Index, Cranial-Arterial Pressure Index (ICP/(MAP - ICP)); CSF, cerebrospinal fluid; ICP, intracranial pressure.
